# Первичный гиперпаратиреоз у детей

**DOI:** 10.14341/probl13382

**Published:** 2023-10-15

**Authors:** А. Р. Бенина, А. А. Колодкина, А. Н. Тюльпаков, Н. Ю. Калинченко, Д. Н. Бровин, А. В. Аникиев, О. С. Даниленко, М. С. Шеремета, В. В. Захарова, Е. Н. Солодовникова, О. Б. Безлепкина

**Affiliations:** Национальный медицинский исследовательский центр эндокринологии; Национальный медицинский исследовательский центр эндокринологии; Медико-генетический научный центр имени академика Н.П. Бочкова; Российская детская клиническая больница ФГАОУ ВО РНИМУ им. Н.И. Пирогова Минздрава России; Национальный медицинский исследовательский центр эндокринологии; Национальный медицинский исследовательский центр эндокринологии; Национальный медицинский исследовательский центр эндокринологии; Национальный медицинский исследовательский центр эндокринологии; Национальный медицинский исследовательский центр эндокринологии; Национальный медицинский исследовательский центр эндокринологии; Национальный медицинский исследовательский центр эндокринологии; Национальный медицинский исследовательский центр эндокринологии

**Keywords:** первичный гиперпаратиреоз, гиперкальциемия, дети, генетическое исследование, аденома паращитовидной железы

## Abstract

**ОБОСНОВАНИЕ:**

ОБОСНОВАНИЕ. Первичный гиперпаратиреоз (ПГПТ) — эндокринное заболевание, характеризующееся избыточной секрецией паратиреоидного гормона (ПТГ) при верхне-нормальном или повышенном уровне кальция крови вследствие первичной патологии околощитовидных желез (ОЩЖ). ПГПТ является редкой патологией для детей, распространенность составляет 2–5:100 000 детского населения. В связи с неспецифичностью клинических проявлений в дебюте (тошнота, рвота, боли в животе, эмоциональная лабильность) заболевание может длительное время оставаться не диагностированным.

**ЦЕЛЬ:**

ЦЕЛЬ. Изучить особенности течения и молекулярно-генетическую основу первичного гиперпаратиреоза у детей.

**МАТЕРИАЛЫ И МЕТОДЫ:**

МАТЕРИАЛЫ И МЕТОДЫ. Ретроспективное наблюдательное исследование 49 пациентов с диагнозом «Первичный гиперпаратиреоз». Всем проведено комплексное лабораторно-инструментальное и молекулярно-генетическое исследование в Институте детской эндокринологии ФГБУ «НМИЦ эндокринологии» Минздрава России в период 2014–2022 гг.

**РЕЗУЛЬТАТЫ:**

РЕЗУЛЬТАТЫ. Первые клинические симптомы ПГПТ отмечались в возрасте 13,8 года [10,6; 15,2], среди которых наиболее часто встречались утомляемость, головные боли, диспепсия, боли в нижних конечностях, переломы. Возраст постановки диагноза составил 15,81 года [13,1; 16,8], у всех детей выявлен высокий уровень ПТГ, общего и иони­зированного кальция, при этом гипофосфатемия была у 93,9% пациентов (n=46), гиперкальциурия у 43% (n=21). У 5 из 49 пациентов (10,2%) выявлена эктопия ОЩЖ: 3 пациента — интратиреоидное расположение ОЩЖ и 2 пациента — эктопия в средостении. При проведении молекулярно-генетического исследования патогенные варианты выявлены у 32,7% пациентов (n=16, ДИ (21; 47)), наиболее часто встречались мутации в гене MEN1 (n=11). У 3 пациентов выявлены патогенные варианты в гене CDC73, в 2 случаях — в гене RET. Прооперировано 39 пациентов, аденома ОЩЖ выявлена в 84,6% случаев (n=33), гиперплазия — в 7,7% (n=3), атипическая аденома — в 5,1% (n=2), карцинома — в 5,1% случаев (n=2).

**ЗАКЛЮЧЕНИЕ:**

ЗАКЛЮЧЕНИЕ. В работе представлены особенности течения и результаты молекулярно-генетического исследования ПГПТ у детей. Данная выборка является самой крупной среди опубликованных в Российской Федерации.

## Abstract (en)

**BACKGROUND:**

BACKGROUND: Primary hyperparathyroidism (PHPT) is an endocrine disorder characterized by excessive secretion of parathyroid hormone (PTH) with upper-normal or elevated blood calcium levels due to primary thyroid gland pathology. PHPT is a rare pathology in children, with a prevalence of 2–5:100,000 children according to the literature. Due to the non-specificity of clinical manifestations at onset (nausea, vomiting, abdominal pain, emotional lability), the disease may remain undiagnosed for a long time.

**AIM:**

AIM: To study the features of the course and molecular genetic basis of primary hyperparathyroidism in children.

**MATERIALS AND METHODS:**

MATERIALS AND METHODS: Retrospective observational study of 49 patients diagnosed with primary hyperparathyroidism. All patients underwent a comprehensive laboratory-instrumental and molecular genetic study at the Institute of Pediatric Endocrinology, Endocrinology Research Center of Russia in the period 2014–2022.

**RESULTS:**

RESULTS: The first clinical symptoms of PHPT were noted at the age of 13.8 years [10.6; 1 5.2], among which fatigue, headaches, dyspepsia, lower limb pain, and fractures were the most common. The age of diagnosis was 15.81 years [13.1; 16.8], all children were found to have high levels of PTH, total and ionized calcium, with hypophosphatemia in 93.9% of patients (n=46) and hypercalciuria in 43% (n=21). Five out of 49 patients (10.2%) were found to have ectopy of the thyroid: 3 showed an intrathyroidal location, 2 in the mediastinal region. Molecular genetic study revealed mutations in 32.7% of patients (n=16, CI (21; 47)), mutations in MEN1 being the most frequent (n=11). Pathogenic variants in CDC73 were detected in 3 patients, RET — in 2. Among the operated 39 patients, adenoma of the thyroid was detected in 84.6% of cases (n=33), hyperplasia in 7.7% (n=3), atypical adenoma in 5.1% (n=2), carcinoma in 5.1% of cases (n=2).

**CONCLUSION:**

CONCLUSION: The paper presents the peculiarities of the course and the results of molecular genetic study of pediatric PHPT. This sample is the largest among those published in the Russian Federation.

## Обоснование

Первичный гиперпаратиреоз (ПГПТ) — эндокринное заболевание, характеризующееся избыточной секрецией ПТГ при верхне-нормальном или повышенном уровне кальция крови вследствие первичной патологии околощитовидных желез (ОЩЖ) [1]. В общей популяции распространенность ПГПТ составляет около 0,86–1% [1][2]. По результатам анализа 1914 пациентов с ПГПТ (Российский регистр пациентов с первичным гиперпаратиреозом) на декабрь 2017 г. распространенность по Москве составила 13 случаев на 100 000 взрослого населения [1]. У детей ПГПТ является крайне редкой патологией, в работах отечественных авторов имеются немногочисленные описания пациентов [3–8]. По данным зарубежных авторов, распространенность среди детей составляет 2–5:100 000 [9–11].

Около 90–95% случаев ПГПТ являются спорадическими [12]. На долю генетических форм приходится 5–10%, где ПГПТ является одним из компонентов наследственных синдромов, таких как синдром множественных эндокринных неоплазий 1, 2A и 4 типов, синдром гиперпаратиреоза с опухолью челюсти, обусловленные мутациями в генах MEN1, RET, CDKN1B и CDC73 соответственно [12][13][14]. Инактивирующие мутации в гене CASR приводят к развитию тяжелого неонатального гиперпаратиреоза, являющегося жизнеугрожающим состоянием [15][16][17]. В 85–90% случаев ПГПТ отмечается поражение одной ОЩЖ, множественные аденомы или гиперплазии встречаются в 5–10%, и менее 1% случаев представлены карциномой ОЩЖ [1][4][18][19].

Данные о ПГПТ у детей ограничены одноцентровыми исследованиями или отдельными клиническими случаями [3][10][20][21].

В связи с неспецифичностью клинических проявлений в дебюте (тошнота, рвота, боли в животе, эмоциональная лабильность) заболевание может длительное время оставаться не диагностированным [20].

## Цель ИССЛЕДОВАНИЯ

Изучить особенности течения и молекулярно-генетическую основу первичного гиперпаратиреоза у детей.

## МАТЕРИАЛЫ И МЕТОДЫ

## Место и время проведения исследования

Анализ данных проведен у пациентов, проходивших обследование и лечение в Институте детской эндокринологии ФГБУ «НМИЦ эндокринологии» Минздрава России за период с сентября 2014-го по декабрь 2022 г.

## Изучаемые популяции (одна или несколько)

Популяция: пациенты от 10 до 17 лет с первичным гиперпаратиреозом.

Критерии включения: наличие гиперкальциемии на фоне высокого уровня ПТГ.

Критерии исключения: хроническая почечная недостаточность, вторичный и третичный гиперпаратиреоз, опухолевые и гранулематозные заболевания.

## Способ формирования выборки из изучаемой популяции (или нескольких выборок из нескольких изучаемых популяций)

Сплошной способ формирования выборки.

## Дизайн исследования

Одноцентровое ретроспективное одновыборочное исследование, включавшее 49 детей с первичным гиперпаратиреозом.

## Методы

Протокол исследования включал в себя оценку жалоб, подробный сбор анамнеза жизни и заболевания, данных наследственного анамнеза, клинический осмотр с оценкой антропометрических данных и наличия костных деформаций.

Гормональные и биохимические исследования проводились в клинико-диагностической лаборатории ФГБУ «НМИЦ эндокринологии» Минздрава России и включали оценку уровня ПТГ, кальция общего и ионизированного в сыворотке крови; исследование уровня кальция в разовой и/или суточной порциях мочи.

Ультразвуковое исследование (УЗИ) проводилось на ультразвуковом сканере (Voluson E8, GE Healthcare, Австрия) с использованием линейного датчика с частотой 10–12 Мгц и включало поиск образования ОЩЖ в виде дополнительного гипоэхогенного образования в местах типичной/атипичной локализации с интранодулярной васкуляризацией, а также исследование почек и органов брюшной полости для диагностики осложнений заболевания в виде мочекаменной (МКБ) и желчнокаменной болезней (МКБ).

Планарная сцинтиграфия ОЩЖ с Тс-99m-технетрилом (MIBI) и ОФЭКТ-КТ проводилась в отделении радионуклидной терапии ФГБУ «НМИЦ эндокринологии» Минздрава России. Визуализация образований проводилась через 15 и 90 минут после введения внутривенного контраста (Тс-99m-технетрил (MIBI)) с оценкой накопления радиофармпрепарата, а также проведение ОФЭКТ-КТ в позднюю фазу (без контраста).

Мультиспиральная компьютерная томография (МСКТ) органов шеи и средостения с внутривенным контрастированием проводилась в отделении диагностики ФГБУ «НМИЦ эндокринологии» с целью уточнения локализации образований ОЩЖ.

Оценка костных деформаций верхних и/или нижних конечностей проводилась с помощью рентгенографии в прямой проекции по стандартной методике.

Для исследования минеральной плотности костной ткани (МПК) проводилась денситометрия поясничного отдела с использованием аппарата Lunar iDXA с оценкой критерия Z-score (стандартное отклонение фактической плотности кости по отношению к соответствующему средневозрастному показателю), где Z-score до -1 SD — норма, менее -1 SD — остеопения.

Молекулярно-генетическое исследование проводилось в лаборатории генетики моногенных эндокринных заболеваний ФГБУ «НМИЦ эндокринологии» Минздрава России: секвенирование по Сэнгеру гена MEN1 и гена CCND1; секвенирование следующего поколения (NGS) панели генов, содержащей праймеры для мультиплексной ПЦР и секвенирования кодирующих последовательностей генов: AIP, AP2S1, CASR, CDC73, CDKN1A, CDKN1B, CDKN1C, CDKN2A, CDKN2C, CDKN2D, DICER1, FAM111A, GATA3, GCM2, GNA11, GNAS, MEN1, POU1F1, PRKAR1A, PRKCA, PTEN, PTTG2, SDHA, SDHB, SDHC, SDHD, TBCE, RET. Оценка патогенности вариантов нуклеотидной последовательности проводилась согласно международным и российским рекомендациям [22][23].

## Статистический анализ

Расчет данных производился с помощью статистического пакета Statistica 8 (StatSoft inc., США), MS Exel 2016 (Microsoft, США). Количественные результаты представлены в виде медианы (Ме) и квартилей [Q1; Q3], соответствующих 25 и 75 перцентилям. Для оценки достоверности различий между изучаемыми подгруппами использовались критерий Манна-Уитни. Критический уровень значимости различий принимали ≤0,05.

Доверительный интервал (ДИ) 95% для относительных частот рассчитан с помощью метода Клоппера-Пирсона.

## Этическая экспертиза

Проведение исследования одобрено Локальным этическим комитетом ФГБУ «НМИЦ эндокринологии» Минздрава России 12.10.2022 г.

## Результаты

В исследование включено 49 пациентов (23 мальчика, 26 девочек) с ПГПТ, обследованных в период с сентября 2014-го по декабрь 2022 г. Возраст проявления первых симптомов ПГПТ составил 13,8 года [ 10,6; 15,2]. Медиана возраста установки диагноза ПГПТ составила 15,8 года [ 13,1; 16,8]. Наиболее частыми клиническими симптомами при постановке диагноза были утомляемость, головные боли, диспепсия, боли в нижних конечностях, 16,3% пациентов имели в анамнезе переломы (рис. 1). У 12 детей (24,5%) жалоб на момент постановки диагноза не отмечалось, и основанием для обследования 3 из них был отягощенный семейный анамнез по МЭН1, у 5 — выявленное в ходе УЗИ по месту жительства образование щитовидной железы, у 3 — случайно выявленная гиперкальциемия и у 1 — гиперкальциурия.

**Figure:**
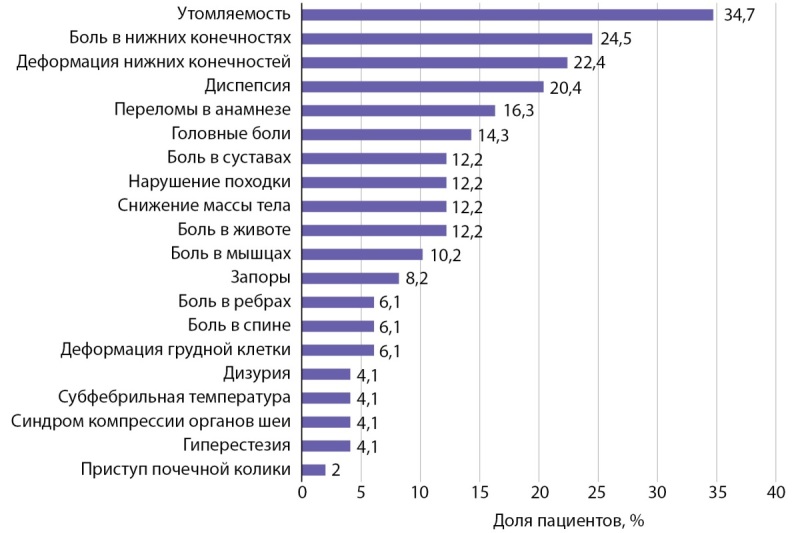
Рисунок 1. Частота встречаемости симптомов на момент постановки диагноза.

Уровни гормонально-биохимических показателей у детей на момент диагностики заболевания представлены в табл. 1. У всех пациентов отмечалось повышение ПТГ, общего и ионизированного кальция. Гипофосфатемия выявлена у 93,9 % пациентов (n=46), гиперкальциурия — у 43% детей (n=21) ДИ (30; 57). Гендерных различий ни по одному из лабораторных параметров выявлено не было.

**Table:** Таблица 1. Лабораторные показатели у детей с ПГПТ на момент постановки диагноза

Параметры	Число пациентов	Медиана	Референсные значения
ПТГ (пг/мл)	49	148,1 [ 87,0; 532,9]	15–65
Общий кальций крови (ммоль/л)	49	2,97 [ 2,73; 3,2]	2,1–2,55
Ионизированный кальций крови (ммоль/л)	49	1,37 [ 1,3; 1,49]	1,03–1,29
Фосфор (ммоль/л)	49	1,05 [ 0,91; 1,26]	1,45–1,78
Кальций в разовой порции мочи (ммоль/л)	23	8,35 [ 4,44; 11,5]	1,7–5,3
Кальций в суточной порции мочи (ммоль/сут)	26	7,6 [ 4,6; 10]	2,5–8

Длительная гиперкальциемия является причиной поражения органов-мишеней. На момент обследования ЖКБ выявлена у 2 пациентов, МКБ у 14. С диагнозом «Гастрит» наблюдались 19 детей, у 4 пациентов были эпизоды острого панкреатита. Денситометрия проведена 25 пациентам, у 19 из них выявлено снижение МПК.

УЗИ по месту жительства было проведено у 30 пациентов, среди которых только у 17 выявлена патология ОЩЖ, а у 9 пациентов образование ОЩЖ было расценено как узел щитовидной железы (ЩЖ).

По результатам УЗИ в ФГБУ «НМИЦ эндокринологии» у 43 пациентов (93,5%) выявлено изменение одной ОЩЖ, множественные поражения выявлены у 3 обследованных, из них у 2 пациентов — образования трех ОЩЖ, у 1 — изменение двух ОЩЖ. У 3 пациентов изменений ОЩЖ, по данным УЗИ, выявлено не было. Наиболее часто новообразования отмечались в области левой нижней (n=18, 40,9%) и правой нижней ОЩЖ (n=16, 36,4%). Образования левой верхней ОЩЖ встречались в 25% случаев (n=11), правой верхней в 13,6% (n=6).

Сцинтиграфия с ОФЭКТ-КТ проведена 33 пациентам, среди них у 26 человек данные сцинтиграфии совпали с результатами УЗИ. У 2 детей по результатам сцинтиграфии выявлены множественные образования ОЩЖ, которые не визуализировались на УЗИ. У 5 пациентов выявлено эктопическое расположение образования ОЩЖ (табл. 2): у 3 пациентов образование было локализовано в ткани щитовидной железы (табл. 2, рис. 2), у 2 — в средостении. У одного из них (пациент №3, рис. 3) данные УЗИ были интерпретированы как образования нижних ОЩЖ, однако при выполнении сцинтиграфии аденома располагалась в области средостения.

**Table:** Таблица 2. Локализация эктопированных образований ОЩЖ при различных методах топической диагностики

Пациенты	УЗИ	Сцинтиграфия с Тс-99m-технетрилом с ОФЭКТ-КТ	Мультиспиральная компьютерная томография
1	В правой доле ЩЖ	Средняя треть правой доли ЩЖ	Не проводилась
2	Не визуализировалась	Центральный отдел средостения	Передний отдел нижнего средостения
3	Аденома левой нижней ОЩЖ (?);гиперплазия правой нижней ОЩЖ (?)	Верхнее средостение слева	В переднем средостении в паренхиме тимуса
4	В левой доле ЩЖ	Средняя треть левой доли ЩЖ	Не проводилась
5	В правой доле ЩЖ	В правой доле ЩЖ	Не проводилась

**Figure:**
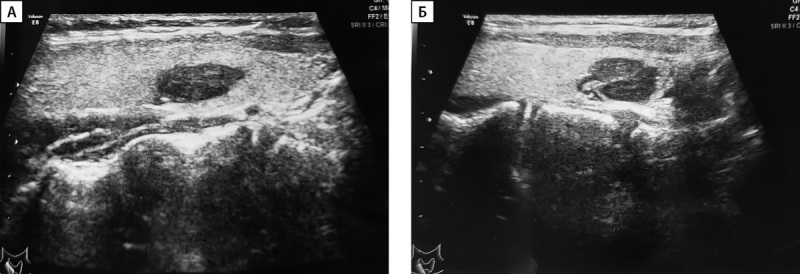
Рисунок 2 (А, Б). Ультразвуковое исследование аденом околощитовидных желез, эктопированных в ткань щитовидной железы.А — пациент №1; Б — пациент №5.

**Figure:**
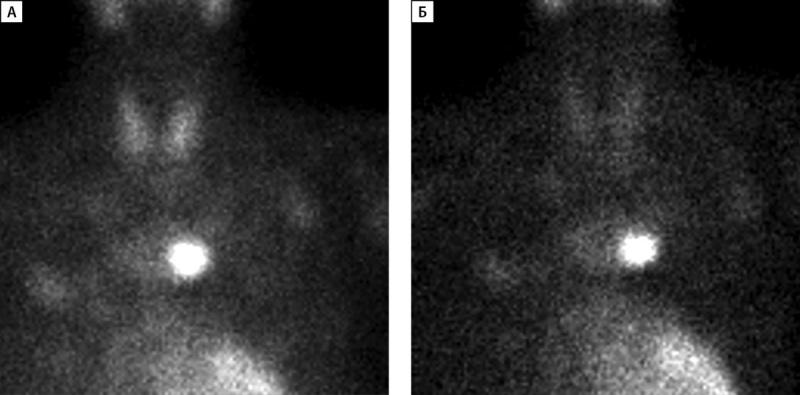
Рисунок 3 (А, Б). Планарная сцинтиграфия образования околощитовидной железы, эктопированного в средостение.А — Через 15 минут после введения контраста; Б — через 90 минут после введения контраста.

В качестве дополнительного метода диагностики у 10 пациентов проведена МСКТ, данные совпали с результатами сцинтиграфии.

Отягощенный наследственный анамнез по МЭН1 был у 8 детей — 16,3% (ДИ 8; 29), 2 пациента имели родственников с ПГПТ.

Секвенирование гена MEN1 проведено 49 пациентам, секвенирование NGS панели генов проведено 22 пациентам. Генетическая основа заболевания установлена у 16 пациентов (32,7% ДИ (21; 47)) (табл. 3). Среди них варианты в гене MEN1 выявлены у 11 пациентов (22%), в гене CDC73 — у 3 (6%), в RET — у 2 (4%). В последние годы изучается роль гена CCND1 в развитии ПГПТ, в связи с чем проведено его исследование у 10 детей — мутации не выявлены.

**Table:** Таблица 3. Молекулярно-генетическое исследование пациентов с ПГПТ Примечание. * — на основании ASMG (American College of Medical Genetics and Genomics) и российского руководства по интерпретации МПС; Het — гетерозиготный вариант, П — патогенный; ВП — вероятно патогенный; НКЗ — неопределенная клиническая значимость.

№ пациента	Ген (транскрипт)	Положение кДНК (замена аминокислотного остатка), HG38	Тип варианта*	Зиготность	Описан/не описан
1	MEN1	NM_130799.2:c.628_631delACAG (p.Thr210Serfs*13)	П	Het	описан
2	MEN1	NM_130799.2:c.666C>A(p.Tyr222*)	П	Het	описан
3	MEN1	NM_130799.2:c.1252G>T(p.Asp418Tyr)	П	Het	описан
4	MEN1	NM_130799.2:с.202_206delGCCCC (p.Ala68Argfs*47)	П	Het	не описан
5	MEN1	NM_130799.2:с.744С>G(р.Ile248Мet)	НКЗ	Het	не описан
NM_130799.2:с.734_745delCTTCCATTGACC (р.Рro245_Asp248del)	НКЗ	не описан
6	MEN1	NM_130799.2:с.1546delC (p.Arg516Glyfs*43)	П	Het	описан
7	MEN1	NM_130799.2:c.1340_1350delTTGAGGGACAG (р.Phe447Cysfs*80)	ВП	Het	не описан
8	MEN1	NM_130799.2:c.1646delC (p.Pro549Glnfs*10)	ВП	Het	не описан
9	MEN1	NM_130799.2:c.959C>G (p.Pro320Arg)	П	Het	описан
10	MEN1	NM_130799.2:c.398_436del (p.Tyr133_Ser145del)	ВП	Het	не описан
11	MEN1	NM_130799.2:с.654+1G>A	П	Het	описан
12	RET	NM_020975.6:c.1901G>A (p.Cys634Tyr)	П	Het	описан
13	RET	NM_020975.6:c.2556C>G (p.Ile852Met)	НКЗ	Het	описан
14	CDC73	NM_024529.5:c.1418delT (p.Ala475Profs*3)	П	Het	не описан
15	CDC73	NM_024529:с.176С>Т (р.Ser59Phe)	НКЗ	Het	описан
16	CDC73	NM_024529:c.78delC (p.Phe27Serfs*10)	П	Het	не описан

Среди всех обследованных паратиреоидэктомия проведена 39 пациентам. По результатам морфологического исследования удаленных ОЩЖ выявлено: 33 аденомы (84,6%), 3 гиперплазии ОЩЖ (7,7%), 2 атипические аденомы (5,1%) и 2 карциномы (5,1%). Сопоставление морфологического и молекулярно-генетического исследования пациентов с ПГПТ представлено в табл. 4.

**Table:** Таблица 4. Молекулярно-генетические и морфологические сопоставления у пациентов с ПГПТ Примечание. n — количество образований.

Морфология ОЩЖ	Ген (транскрипт)	Положение кДНК (замена аминокислотного остатка)	Число пациентов
Аденома (n=33)	MEN1(NM_130799.2)	c.628_631delACAG(p.Thr210Serfs*13)	8
c.666C>A(p.Tyr222*)
c.1252G>T(p.Asp418Tyr)
с.202_206delGCCCC(p.Ala68Argfs*47)
RET(NM 020975.6)	c.1901G>A(p.Cys634Tyr)
c.2556C>G(p.Ile852Met)
CDC73(NM_024529)	с.176С>Т(р.Ser59Phe)
c.1418delT(p.Ala475Profs*3)
Мутации не выявлены	25
Атипическая аденома (n=2)	Мутации не выявлены	2
Гиперплазия (n=3)	MEN1(NM_130799.2)	с.654+1G>A	2
с.202_206delGCCCC(p.Ala68Argfs*47)
Мутации не выявлены	1
Карцинома (n=2)	CDC73(NM_024529)	c.78delC(p.Phe27Serfs*10)	1
Мутации не выявлены	1

## Обсуждение

Первичный гиперпаратиреоз является редкой патологией в детском возрасте. Медиана возраста начала клинических проявлений в нашей группе составила 13,8 [ 10,6; 15,2] года, диагноз был установлен в среднем через 2 года, что может быть связано с неспецифичностью клинических проявлений, таких как утомляемость, диспепсия, головные боли. Медиана возраста диагностики ПГПТ в проведенном исследовании составила 15,8 [ 13,1; 16,8] года, что сопоставимо с работами зарубежных авторов, таких как Josh Kollars et al. и Wenbo Wang et al., где возраст диагностики ПГПТ варьировал от 14 до 17 лет [10][20].

В 85–90% случаев ПГПТ обусловлен поражением одной ОЩЖ, у 5–10% пациентов изменениям подвергаются несколько желез [1][18]. В проведенном исследовании изолированная аденома выявлена у 93,5% пациентов (n=43), множественное поражение ОЩЖ отмечалось в 6% случаев (n=3). У всех пациентов множественные образования ОЩЖ были обусловлены мутациями в гене MEN1.

В работах Hani Z. et al. и Sirmen Kızılcan Çetin et al. [24][25] наиболее часто встречались изменения нижних ОЩЖ, что отмечалось и в нашем исследовании: у 18 детей (40,9%) имелось образование левой нижней ОЩЖ, у 16 (36,4%) — правой нижней.

Ввиду анатомических особенностей топическая диагностика аденом ОЩЖ в ряде случаев может быть затруднена. В проведенном исследовании у 9 пациентов при выполнении УЗИ на амбулаторном этапе по месту жительства образование ОЩЖ было расценено как узел ЩЖ. В своей работе Гостимский А.В. и соавт. отмечали, что часто УЗИ является неинформативным для определения точной локализации образований ОЩЖ [3]. Особую трудность диагностики представляют эктопические образования ОЩЖ. По данным литературы, распространенность эктопий ОЩЖ в детской популяции варьирует от 5 до 26% [26]. Наиболее частыми локализациями являются паренхима тимуса и ткань ЩЖ, реже встречается эктопия в пространстве около пищевода и средостения [27]. В настоящем исследовании эктопия ОЩЖ отмечалась в ткани ЩЖ (n=3), в области средостения (n=1) и паренхиме тимуса (n=1).

У одного из пациентов (табл. 2, пациент №3) при проведении УЗИ ОЩЖ выявлены образования левой и правой нижних ОЩЖ, однако, по результатам сцинтиграфии и МСКТ, образование ОЩЖ располагалось в области средостения (рис. 3). Таким образом, сцинтиграфия с ОФЭКТ-КТ, а в некоторых случаях и МСКТ являются предпочтительными методами визуализации образований ОЩЖ.

На долю генетических форм ПГПТ приходится около 5–10%, среди которых наиболее распространенными являются варианты в гене MEN1 [28][29]. В нашем исследовании патогенные варианты выявлены в 32,7% случаев (n=16), самые частые — в гене MEN1 (n=11). Варианты в CCND1 встречаются при спорадических аденомах ОЩЖ [30]. Ген кодирует циклин D1, участвующий в регуляции клеточного цикла [29]. В проведенном исследовании CCND1 исследован у 10 пациентов — патогенные варианты не найдены, что, вероятно, связано с небольшой выборкой изучаемых пациентов.

Ген CDC73 (известный также как HRPT2) кодирует белок парафибромин и до 80% случаев ассоциирован со спорадической карциномой ОЩЖ [31]. Варианты в CDC73 приводят к развитию редкого синдрома гиперапаратиреоза с опухолью челюсти с аутосомно-доминантным наследованием, включающего в себя оссифицирующие опухоли челюсти, опухоли матки и кисты почек [30]. Синдром наиболее часто ассоциируется с карциномами ОЩЖ [30]. В работе A. Rahimi et al. представлены обобщенные данные, опубликованные с 1972-го по 2020 гг. о 18 случаях карцином ОЩЖ у детей, среди которых отмечались варианты в CDC73 [32]. В нашем исследовании патогенные варианты в CDC73 выявлены у 3 пациентов. На момент обследования дополнительных компонентов синдрома выявлено не было (только ПГПТ), что требует дальнейшего динамического наблюдения. В проведенном исследовании среди оперированных пациентов у 2 (5,4% ДИ (0; 19)) выявлена карцинома ОЩЖ, одна из которых обусловлена патогенным вариантом в CDC73.

Синдром МЭН2А, в рамках которого встречается ПГПТ, развивается вследствие мутаций в гене RET, кодирующем протонкоген. Основными компонентами синдрома являются медуллярный рак щитовидной железы (МРЩЖ) и феохромоцитома [33]. ПГПТ при МЭН2А развивается в 10–30% случаев и имеет легкое течение [33]. В проведенном исследовании варианты в гене RET выявлены у 2 человек. У одного пациента, помимо ПГПТ, диагностирован МРЩЖ, обусловленный патогенным вариантом c.1901G>A (p.Cys634Tyr). У другого пациента выявлен гетерозиготный вариант c.2556C>G (p.Ile852Met) с неопределенной клинической значимостью (НКЗ), дополнительных компонентов синдрома выявлено не было. В статье Andreas Machens et al. описана семья с вариантом p.Ile852Met в RET [34]. У пациентов не отмечалось признаков феохромоцитомы или ПГПТ, однако некоторые из них имели достоверное повышение кальцитонина крови [34], что требует динамического наблюдения.

Удаление образований ОЩЖ является единственным радикальным методом лечения ПГПТ. При проведении морфологического исследования наиболее часто выявлялись аденомы ОЩЖ (84,6%). Особый интерес представляют атипические аденомы (АА) ОЩЖ, которые имеют промежуточное морфологическое строение между аденомой и карциномой ОЩЖ и обладают неопределенным злокачественным потенциалом [35][36]. Распространенность АА составляет от 0,5 до 4,4% случаев среди оперированных пациентов по поводу ПГПТ [37]. В отличие от аденомы, атипическая аденома может иметь ряд морфологических характеристик, схожих с карциномой, при этом не соответствовать абсолютным критериям злокачественности, таким как инвазивный рост в окружающие ткани, сосудистая инвазия, высокая митотическая активность (>5/10), фиброзные тяжи, некроз опухоли, клеточная атипия [35][36]. Атипическая аденома отличается от рака ОЩЖ отсутствием однозначной инвазии в капсулу на всю толщину, сосудистой/периневральной или инвазией в соседние структуры [37]. В настоящем исследовании АА ОЩЖ выявлена у двух детей. Среди морфологических особенностей отмечались: широкие фиброзные септы, митотическая активность (3/10), отсутствие инвазивного роста. Дифференциация АА от карциномы имеет особые трудности. Для наиболее точной диагностики необходимо динамическое наблюдение.

## ЗАКЛЮЧЕНИЕ

ПГПТ у детей — редкая патология. Ввиду неспецифичности клинических проявлений заболевание диагностируется у детей в среднем через 2 года после возникновения первых симптомов. Для выявления образований ОЩЖ необходимо проведение сцинтиграфии с ОФЭКТ-КТ, что позволит наиболее точно определить локализацию образования, особенно это касается эктопических форм. Для определения причины ПГПТ, а также для исключения синдромальной патологии необходимо проведение молекулярно-генетического анализа у детей.

## Ограничения исследования

В ходе исследования могли возникнуть смещения результатов по причине недостаточного объема выборки в связи с низкой встречаемостью заболевания.

## Дополнительная информация

Источники финансирования. Работа выполнена при содействии Фонда поддержки и развития филантропии «КАФ».

Конфликт интересов. Авторы декларируют отсутствие конфликта интересов.

Участие авторов. Бенина А.Р., Колодкина А.А., Безлепкина О.Б., Калинченко Н.Ю., Шеремета М.С. — поисково-аналитическая работа и подготовка финальной версии статьи; Бровин Д.Н., Аниеев А.В. — проведение оперативного лечения; Тюльпаков А.Н., Захарова В.В., Солодовникова Е.Н. — проведение молекулярно-генетического исследования, редактирование текста.

Все авторы одобрили финальную версию статьи перед публикацией, выразили согласие нести ответственность за все аспекты работы, подразумевающую надлежащее изучение и решение вопросов, связанных с точностью или добросовестностью любой части работы.
